# Clinical outcomes after a single induction dose of etomidate versus ketamine for emergency department sepsis intubation: a randomized controlled trial

**DOI:** 10.1038/s41598-023-33679-x

**Published:** 2023-04-19

**Authors:** Winchana Srivilaithon, Atidtaya Bumrungphanithaworn, Kiattichai Daorattanachai, Chitlada Limjindaporn, Kumpol Amnuaypattanapon, Intanon Imsuwan, Nipon Diskumpon, Ittabud Dasanadeba, Yaowapha Siripakarn, Thosapol Ueamsaranworakul, Chatchanan Pornpanit, Vanussarin Pornpachara

**Affiliations:** 1 Department of Emergency Medicine, Faculty of Medicine, Thammasat University, 99/209 Phahon Yothin Road, Klong Luang District, Pathum Thani, 12120 Thailand; 2 Division of Endocrinology, Department of Medicine, Rajavithi Hospital, Bangkok, Thailand

**Keywords:** Adverse effects, Infectious diseases, Outcomes research

## Abstract

Patients with sepsis often require emergency intubation. In emergency departments (EDs), rapid-sequence intubation with a single-dose induction agent is standard practice, but the best choice of induction agent in sepsis remains controversial. We conducted a randomized, controlled, single-blind trial in the ED. We included septic patients who were aged at least 18 years and required sedation for emergency intubation. Patients were randomly assigned by a blocked randomization to receive 0.2–0.3 mg/kg of etomidate or 1–2 mg/kg of ketamine for intubation. The objectives were to compare the survival outcomes and adverse events after intubation between etomidate and ketamine. Two hundred and sixty septic patients were enrolled; 130 patients/drug arm whose baseline characteristics were well balanced at baseline. In the etomidate group, 105 patients (80.8%) were alive at 28 days, compared with 95 patients (73.1%) in the ketamine group (risk difference [RD], 7.7%; 95% confidence interval [CI], − 2.5 to 17.9%; P = 0.092). There was no significant difference in the proportion of patients who survived at 24 h (91.5% vs. 96.2%; P = 0.097) and survived at 7 days (87.7% vs. 87.7%; P = 0.574). A significantly higher proportion of the etomidate group needed a vasopressor within 24 h after intubation: 43.9% vs. 17.7%, RD, 26.2% (95% CI, 15.4 to 36.9%; P < 0.001). In conclusion, there were no differences in early and late survival rates between etomidate and ketamine. However, etomidate was associated with higher risks of early vasopressor use after intubation. Trial registration: The trial protocol was registered in the Thai Clinical Trials Registry (identification number: TCTR20210213001). Registered 13 February 2021—Retrospectively registered, https://www.thaiclinicaltrials.org/export/pdf/TCTR20210213001.

## Introduction

Sepsis is a major medical emergency in emergency departments (EDs) and has a high rate of morbidity and mortality^1^. The incidence of sepsis-induced respiratory failure in the United States is 6–7%^2,3^. Some patients require emergency orotracheal intubation to help optimize their oxygenation and ventilation^1,4^. Rapid sequence intubation (RSI) is considered the method of choice in the ED. However, the best induction agent remains controversial.

Etomidate is a nonbarbiturate hypnotic that is most often used in RSI^5^. It has a short duration of action and causes little cardiovascular depression^6^. Single-dose etomidate can inhibit adrenal mitochondrial 11-β-hydroxylase activity and may induce reversible adrenal insufficiency (AI)^7^. Reversible AI may also be exacerbated in patients with critical illness-related corticosteroid insufficiency, particularly in sepsis and septic shock^8^. However, the clinical significance of this association is unclear. A previous meta-analysis comparing etomidate and alternative induction agents concluded that etomidate was associated with higher rates of mortality in patients with sepsis^9^, but more recent studies have reported conflicting results^10,11^.

Ketamine is an alternative induction agent in sepsis. It increases blood pressure and heart rate through catecholamine release and is considered a safe and valuable alternative to etomidate for emergency intubation in patients with sepsis^12^. However, several studies suggest that ketamine may be associated with a greater risk of hypotension than etomidate, especially in patients with catecholamine depletion^5,11,13^. Furthermore, there is a concern that ketamine may be related to increased myocardial ischaemia, especially in elderly patients^14^.

There is no consensus on which induction agent is preferred for emergency intubation in sepsis. A recent meta-analysis suggested that single-dose etomidate, compared to alternative induction agents, was not associated with increased mortality in patients with sepsis. However, the finding might be subject to bias and confounding^11^. Therefore, we conducted a randomized trial that aimed to compare the survival and peri-intubation adverse events after single-dose induction between etomidate and ketamine.

## Methods

### Trial design and oversight

From March 2019 to December 2020, this single-centre, randomized, single-blind, controlled trial, with 1:1 allocation, was conducted by the Emergency Medicine Research Group at Thammasat University Hospital (TUH) in Pathum Thani, Thailand. TUH is an 800-bed tertiary academic teaching hospital in the suburbs north of Bangkok, with approximately 1.1 million people living in the area. The ED of TUH sees 60,000 patients annually, and approximately 500 patients need emergency intubation each year. A previous study showed a very high success rate of emergency intubation overall and at the first attempt rate in the ED of TUH (99.4% and 74.7%, respectively)^15^.

### Ethics approval and consent to participate

The trial was approved by the Human Research Ethics Committee of the Faculty of Medicine of Thammasat University. Because of the sudden and life-threatening nature of cases in patients who needed emergency intubation, the process of obtaining written informed consent was deferred until after the emergency had passed. We sought written informed consent as soon as practicable after the intubation had passed to continue data collection from the patient or, if the patient was unable to give consent, a patient’s legally authorized representative was informed and gave consent for participation in the research. If the patient regain or develop the capacity to consent, then the patient consent was obtained before any further data collection. All experiments were performed in accordance with approved clinical trial protocols and regulations.

### Trial registration

The trial protocol was registered in the Thai Clinical Trials Registry (identification number; TCTR20210213001). Registered 13 February 2021—Retrospectively registered, https://www.thaiclinicaltrials.org/export/pdf/TCTR20210213001.

### Patient population

Patients presenting to the ED with suspected sepsis who were 18 years or older and then needed an induction agent for emergency intubation in the ED were eligible for inclusion. Exclusion criteria were as follows: (1) cardiac arrest before intubation, (2) presence of a do-not-resuscitate order, (3) known or suspected adrenal insufficiency, (4) severe hypertension (blood pressure before randomization: over 180/110 mmHg), and (5) suspected or evidenced increased intracranial pressure. There were no exclusions after application of the randomization criteria in the trial.

### Randomization and treatment

Patients were randomly assigned to receive a single-dose induction agent consisting of either etomidate (Lipuro, B. Braun Melsungen, Germany) administered as a 0.2–0.3 mg/kg intravenous bolus or ketamine (Ketalar, PAR Pharmaceutical, Ireland) administered as a 1–2 mg/kg intravenous bolus. The randomization sequence was determined using a computer-generated randomization table with a block size of four by a statistician who was not involved in determining the patient eligibility, drug administration, intubating procedure, or outcome assessment. The drug allocation sequence was kept inaccessible to the research team throughout the study period. Patient assignments were placed into sequentially numbered sealed opaque envelopes. The emergency physician enrolling patients was responsible for opening these envelopes and preparing the study agent but was not involved in the intubation process. None of the emergency physicians enrolling patients were members of the staff in the inpatient ward, and they had no influence on the management of the patients after they were admitted to the hospital.

All patients received the same standard RSI protocol, except for the single-dose induction agent. The use of a neuromuscular blocking agent immediately after induction (succinylcholine as a 1.5 mg/kg intravenous bolus) depended on the clinical state of the patient and the presence of any contraindications. Patients were intubated by either the direct laryngoscopy technique (Macintosh) or video laryngoscopy technique (GlideScope). Intratracheal tube positioning was confirmed by clinical assessments and capnometers with capnographs.

The definition of sepsis was based on the Third International Consensus Definitions for Sepsis and Septic Shock^16^. Patients in both groups received the same standard therapy in accordance with International Surviving Sepsis Campaign guidelines^17^, including respiratory support, fluid resuscitation, early antimicrobials, and macro- and microcirculation management.

### Outcomes

The primary outcome was 28-day survival. The secondary outcomes were 24-h survival, 7-day survival, early haemodynamic parameters after intubation, amount of fluid required in the first three hours, and occurrence of peri-intubation adverse events. Peri-intubation adverse events included cardiac arrest (during or immediately after intubation), failed intubation, postintubation hypotension (systolic blood pressure below 90 mmHg, or mean arterial blood pressure below 65 mmHg), and use of a vasopressor (norepinephrine, epinephrine, or dopamine) within the first 24 h after intubation. Outcomes were assessed by trained research coordinators who were unaware of treatment assignment.

### Sample size estimation

A pilot study was performed to obtain the preliminary data for the calculation of a sample size for the primary outcome. Our power was determined by the survival rate of the pilot population. We determined that a group of 130 patients in the etomidate allocation and a group of 130 patients in the ketamine allocation were needed to detect ten percent absolute risk differences with 80% power and a type-I error of 0.05.

### Statistical analysis

The independent data monitoring committee performed an interim analysis every 6 months. We used the Haybittle-Peto boundary to determine the upper and lower stopping boundaries for the primary outcome, with no adjustment in the final analysis.

The survival outcomes were analysed without adjustment in the intention-to-treat population, which included all the patients who were randomized. All included patients were confirmed to have received the assigned intervention. Trial data were summarized by the calculation of means and standard deviations for normally distributed variables, median and interquartile ranges for nonnormally distributed variables, and frequency and percentage for categorical variables. The magnitude of the difference between two percentages was demonstrated by the risk difference with 95% confidence intervals. All statistical tests were two-sided. A p value less than 0.05 was considered statistically significant. All analyses were performed using STATA software version 14.0 (StataCorp, College Station, TX).

## Results

### Patients and interventions

**Figure 1:**
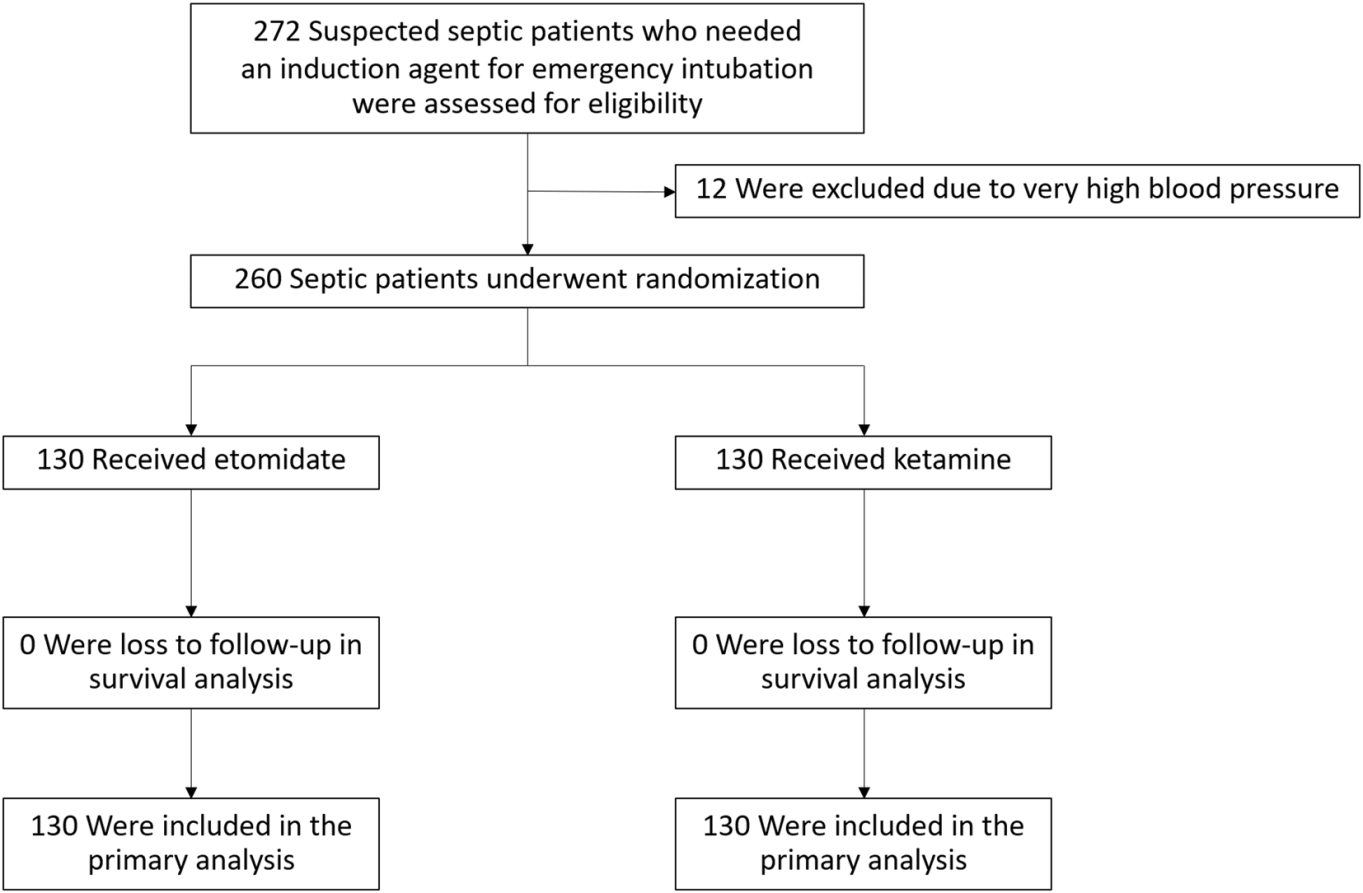
Flow diagram of the study patients enrolled.

A total of 272 suspected sepsis patients who needed an induction agent for emergency intubation were enrolled. 12 patients were excluded because of very high blood pressures before intubation (Fig. 1). The remaining 260 patients with sepsis underwent randomization and were followed up for 28 days (130 patients in the etomidate group and 130 in the ketamine group). The primary outcome was obtained for all patients. The characteristics of the patients were well balanced at baseline (Table 1). The physiological parameters before intubation were also similar in the two groups. The key predictors of mortality in sepsis (delta SOFA score and initial serum lactate) were also similar in the two groups.

**Table 1:** Characteristics of the patients at baseline.

Characteristic	All patients (N = 260) n (%)	Etomidate (N = 130) n (%)	Ketamine (N = 130) n (%)
Male gender	153 (58.9)	77 (59.2)	76 (58.5)
Age, mean (± SD) (years)	71.9 (± 13.9)	73.2 (± 12.6)	70.5 (± 14.9)
Comorbid disease			
Diabetic mellitus	107 (41.2)	48 (36.9)	59 (45.4)
Hypertension	156 (60.0)	80 (61.5)	76 (58.5)
Stroke	69 (26.5)	43 (33.1)	26 (20.0)
Chronic kidney disease	31 (11.9)	17 (13.1)	14 (10.8)
COPD/asthma	20 (7.7)	12 (9.2)	8 (6.2)
Reasons for emergency intubation			
Acute respiratory failure	107 (41.2)	55 (42.3)	52 (40.0)
Pneumonia	96 (36.9)	46 (35.4)	50 (38.5)
Coma	42 (16.2)	23 (17.7)	19 (14.6)
Shock	7 (2.7)	4 (3.1)	3 (2.3)
Other	8 (3.0)	2 (1.5)	6 (4.6)
Sources of infection			
Respiratory tract	187 (71.9)	95 (73.1)	92 (70.8)
Intra-abdominal	24 (9.2)	12 (9.2)	12 (9.2)
Skin or soft tissue	17 (6.5)	8 (6.2)	9 (6.9)
Urinary tract	13 (5.0)	7 (5.4)	6 (4.6)
Glasgow coma scale before intubation			
14–15	93 (35.8)	42 (32.3)	51 (39.2)
9–13	85 (32.7)	49 (37.7)	36 (27.7)
3–8	82 (31.5)	39 (30.0)	43 (33.1)
Physiological parameters before intubation			
Systolic blood pressure, mean (± SD) (mmHg)	115.5 (± 31.7)	112.9 (± 30.7)	118.1 (± 32.5)
Pulse rate, mean (± SD) (bpm)	107.2 (± 24.9)	108.8 (± 24.5)	105.6 (± 25.2)
Oxygen saturation, median (IQR) (%)	92 (83, 98)	92 (84, 98)	92 (83, 98)
qSOFA score, mean (± SD)	2.2 (± 0.4)	2.2 (± 0.4)	2.1 (± 0.3)
Delta SOFA score at ED, mean (± SD)	4.8 (± 1.9)	4.6 (± 1.9)	4.9 (± 1.9)
Initial serum lactate, median (IQR) (mmol/L)	3.3 (2.4, 6.5)	3.6 (2.4, 7.6)	3.2 (2.2, 5.4)
Receive intravenous antibiotic before randomization	214 (82.3)	108 (83.1)	106 (81.5)
Amount of intravenous fluid before randomization, median (IQR) (mL)	1000 (600, 1500)	1000 (500, 1500)	1200 (650, 1500)

Intubation conditions between the two groups were also similar (Table 2), including the total number of attempts, success at the first attempt, difficult intubation indicators, pretreatment with intravenous fluid, glottic exposure grade, and patients’ physiological parameters after intubation. However, the proportion of patients who received neuromuscular blocking agents during intubation was significantly higher (P = 0.04) in the ketamine group than in the etomidate group (76.9% vs. 64.6%, respectively).

**Table 2:** Intubation conditions of the study patients.

Intubation condition	Etomidate (N = 130) n (%)	Ketamine (N = 130) n (%)	P value
Total number of attempts, median (IQR)	1 (1, 1)	1 (1, 1)	0.579
Successful in the first attempt	116 (89.2)	114 (87.7)	0.846
Failed intubation	2 (1.5)	0	0.498
Difficult intubation indicator			
Large tongue	1 (0.8)	7 (5.4)	0.066
Limited mouth opening	1 (0.8)	1 (0.8)	1.000
Short hypo-mental distance	3 (2.3)	3 (2.3)	1.000
Short thyro-hyoid distance	2 (1.5)	5 (3.9)	0.447
Poor neck mobility	1 (0.8)	2 (1.5)	1.000
Pretreatment with intravenous fluid	42 (32.3)	40 (30.8)	0.894
Neuromuscular blocking agent used	84 (64.6)	100 (76.9)	0.040
Glottis exposure grade			0.346
I = Visualized entire vocal cord	68 (52.3)	80 (61.5)	
II = Visualized part of vocal cord	51 (39.2)	41 (31.5)	
III = Visualized epiglottis only	10 (7.7)	9 (6.9)	
IV = non-visualized epiglottis	1 (0.8)	0	
Physiological parameters after intubation			
Systolic blood pressure, mean (± SD) (mmHg)	132.9 (± 46.9)	142.6 (± 37.9)	0.068
Pulse rate, mean (± SD) (bpm)	116.6 (± 23.5)	112.5 (± 21.5)	0.139
Oxygen saturation, median (IQR) (%)	100 (100, 100)	100 (100, 100)	0.021

### Primary and secondary outcomes

**Figure 2:**
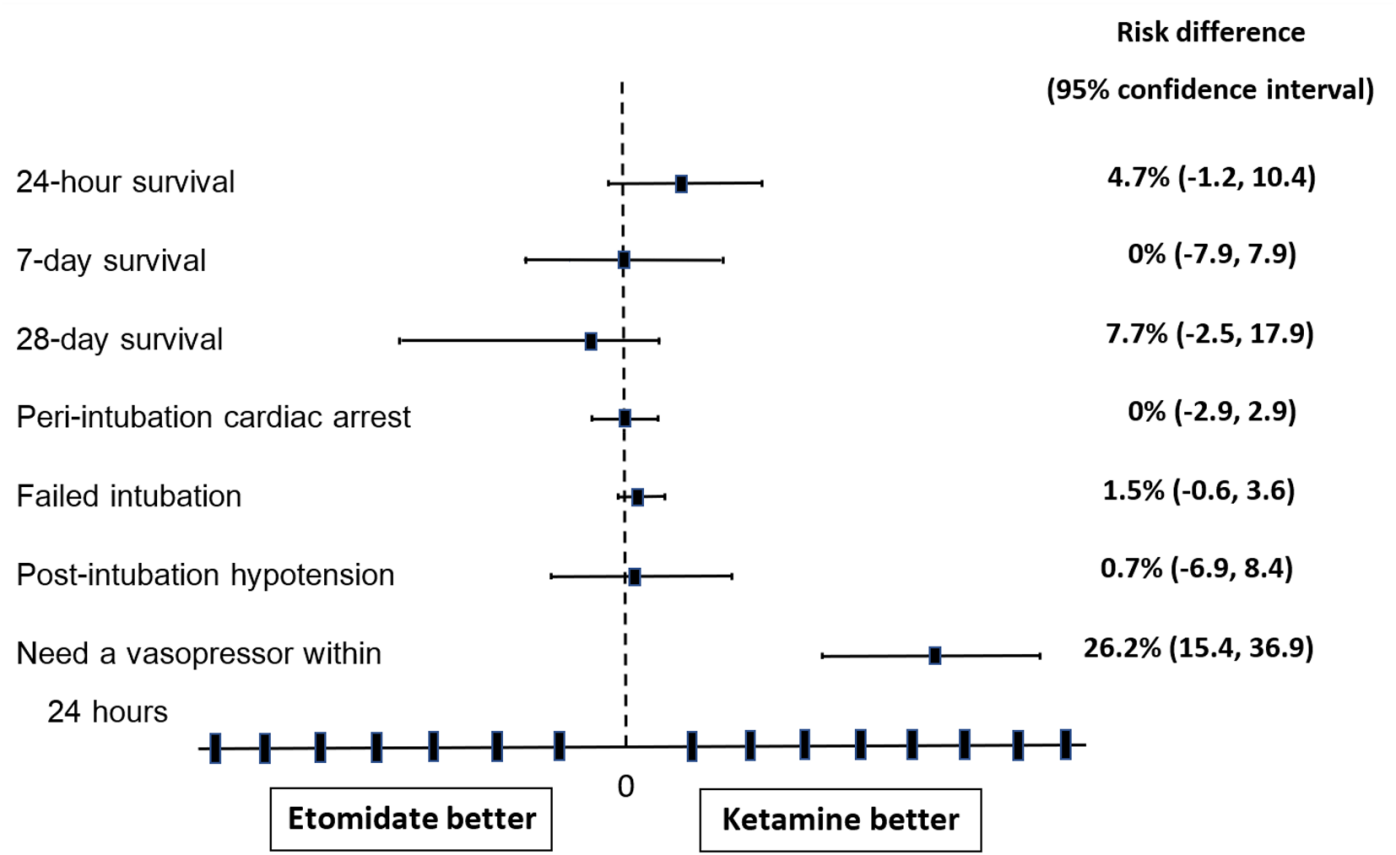
Primary and secondary outcomes.

In the etomidate group, 105 patients (80.8%) were alive at 28 days compared to 95 patients (73.1%) in the ketamine group (risk difference [RD], 7.7%; 95% confidence interval [CI], − 2.5 to 17.9%; P = 0.092). There were no significant differences between the etomidate group and the ketamine group in the proportion of patients who survived at 24 h (91.5% vs. 96.2%, respectively; RD, 4.7%; 95% CI, − 1.2 to 10.4%; P = 0.097) or survival at 7 days (87.7% vs. 87.7%; RD, 0%; 95% CI, − 7.9 to 7.9%; P = 0.574) (Table 3 and Fig. 2).

**Table 3:** Primary and secondary outcomes.

Outcome	Etomidate (N = 130) n (%)	Ketamine (N = 130) n (%)	Risk difference (95% confidence interval) (%)	P value
Survival outcomes				
24-h survival	119 (91.5)	125 (96.2)	4.7 (− 1.2, 10.4)	0.097
7-day survival	114 (87.7)	114 (87.7)	0 (− 7.9, 7.9)	0.574
28-day survival	105 (80.8)	95 (73.1)	7.7 (− 2.5, 17.9)	0.092
Peri-intubation adverse events				
Cardiac arrest	2 (1.5)	2 (1.5)	0 (− 2.9, 2.9)	1.000
Failed intubation	2 (1.5)	0	1.5 (− 0.6, 3.6)	0.155
Post-intubation hypotension	15 (11.5)	14 (10.8)	0.7 (− 6.9, 8.4)	0.843
Total fluid required in the first three hours, median (IQR) ml	1000 (600, 1500)	1000 (600, 1500)	0 (− 153.9, 123.1)	0.827
Used of a vasopressor medication within 24 h after intubation	57 (43.9)	23 (17.7)	26.2 (15.4, 36.9)	< 0.001
Used of intravenous corticosteroid	19 (14.6)	7 (5.4)	9.2 (2.1, 16.4)	0.012

Regarding peri-intubation adverse events, cardiac arrest during intubation occurred in two patients (1.5%) in the etomidate group and two patients (1.5%) in the ketamine group (RD, 0%; 95% CI, − 2.9 to 2.9%; P = 1.0). Two patients (1.5%) in the etomidate group, but none in the ketamine group, were diagnosed with failed intubation. There was no significant difference between the study groups in the proportion of patients with postintubation hypotension (11.5% vs. 10.8%; RD, 0.7%; 95% CI, − 6.9 to 8.4%; P = 0.843); however, there were significant differences between the etomidate group and the ketamine group in the proportion of patients who needed a vasopressor within 24 h after intubation (43.9% vs. 17.7%, respectively; RD, 26.2%; 95% CI, 15.4 to 36.9%; P < 0.001) and the proportion of patients who received intravenous corticosteroids (14.6% vs. 5.4%, respectively; RD, 9.2%; 95% CI, 2.1 to 16.4%; P = 0.012) (Table 3 and Fig. 2).

## Discussion

The main goal of this study was to compare the clinical outcomes between the two induction agents that are most commonly used for emergency intubation in EDs. We found no significant differences in survival at 24 h, 7 days, and 28 days in sepsis patients intubated with etomidate or ketamine. We also found no significant difference in patients’ physiological parameters after intubation and peri-intubation adverse events, including (1) peri-intubation cardiac arrest, (2) failed intubation, and (3) postintubation hypotension. However, there were significant differences between the etomidate group and the ketamine group in the proportion of patients who required a vasopressor within 24 h after intubation and received intravenous corticosteroids.

There is controversy regarding the safety of single-dose etomidate as an induction agent for emergency intubation in patients with sepsis. Several RCTs compared mortality outcomes between etomidate and alternative induction agents^12,18–20^, but most of them included a broad range of critically ill patients or trauma cases; patients with sepsis were only a subgroup of the population (15–50%) or were in a secondary analysis. One RCT conducted by Tekwani et al.^21^ to study patients with suspected sepsis who were intubated in the ED, focused mainly on the length of hospital stay and not the patients’ clinical outcomes. Our study was designed to answer this specific controversy by including only patients with suspected sepsis who presented to the ED; it showed that single-dose etomidate was an acceptable choice in patients with sepsis in the ED.

Etomidate can suppress the adrenal synthesis of cortisol by inhibiting 11-β hydroxylase, the enzyme responsible for the conversion of 11-deoxycortisol to cortisol^7^^.^ As a result, adrenal function may be blunted for 4–24 h after a single dose, but inhibition can last up to 72 h^22,23^. Relative AI indicates a lack of adrenocortical reserve and has also been found in patients with septic shock. Therefore, single-dose etomidate for emergency intubation should be used with caution, as it may worsen patient outcomes^22^. A previous meta-analysis from Chan et al.^9^ concluded that using etomidate for RSI was associated with higher rates of AI and 28-day mortality in patients with sepsis. In contrast, a more recent meta-analysis from Gu et al.^10^ indicated that although single-dose etomidate increased the risk of AI, it was not associated with increased overall mortality in patients with sepsis. Our findings support this recent meta-analysis by showing that single-dose etomidate was not associated with a significantly increased risk of mortality in patients with suspected sepsis in the ED.

Previous studies comparing etomidate and ketamine in acutely ill patients showed that there were no differences in major peri-intubation adverse events, including peri-intubation cardiac arrest, change in blood pressure after intubation, and the total volume of intravenous fluid needed after intubation^12,20^. Our results support these findings. However, there is controversy surrounding postintubation hypotension.

Single-dose induction agents can impact patients’ haemodynamic status, especially in critically ill patients who need emergency intubation. Although both etomidate and ketamine are considered haemodynamically stable induction agents, there remain concerns that they might cause postintubation hypotension, particularly in patients with sepsis^5,9,11,24–26^. Our study included all suspected sepsis patients who needed emergency intubation regardless of their cardiovascular status. Only 23% of participants exhibited shock prior to intubation. However, our study showed that approximately 44% of patients who received etomidate developed hypotension and needed vasopressor medication within 24 h after intubation, compared with 18% such patients in the ketamine group. This can be explained by transient adrenal suppression after a single dose of etomidate^22,23^.

Multicentre observational studies have reported that ketamine is associated with higher risks of postintubation hypotension after emergency intubation than alternative agents^5^, including etomidate^11,26^. These findings are supported by Smischney et al.^24^, who studied critically ill patients in 16 ICUs and found less postintubation hypotension with etomidate than with alternative agents. However, a multicentre observational study in the ED reported lower risks of postintubation hypotension in haemodynamically unstable patients when using ketamine than when using midazolam or propofol^25^. In the emergency department, postintubation hypotension might be associated with a higher risk of mortality^27,28^.

## Limitations

Our study was limited by its sample size, and although our results show no difference in patients’ physiological parameters and postintubation hypotension after a single-dose of etomidate or ketamine, we had limited statistical power. Moreover, we did not calculate the sample size to demonstrate the differences in physiologic parameters in the design phase of the trial. However, our study has provided prospectively collected data on 28-day mortality rates that could be used for future high-quality and adequately powered studies comparing the immediate effects of etomidate and ketamine as well as mortality.

## Conclusions

In patients with clinically suspected sepsis who needed emergency intubation in the ED, there was no difference in early and 28-day survival rates between etomidate and ketamine. However, etomidate was associated with higher risks of early vasopressor use after intubation.

## Data Availability

The datasets used and/or analysed during the current study are available from the corresponding author on reasonable request.

## Abbreviations

**ED**: Emergency department
**RSI**: Rapid sequence intubation
**AI**: Adrenal insufficiency
**RCT**: Randomized controlled trial
**ICU**: Intensive care unit
